# *Helicobacter pylori* Mutations Conferring Resistance to Fluoroquinolones and Clarithromycin among Dyspeptic Patients Attending a Tertiary Hospital, Tanzania

**DOI:** 10.1155/2019/8481375

**Published:** 2019-07-01

**Authors:** Hyasinta Jaka, Nele Rüttgerodt, Wolfgang Bohne, Andreas Mueller, Uwe Gross, Christa Kasang, Stephen E. Mshana

**Affiliations:** ^1^Department of Internal Medicine, Gastroenterology and Hepatology Unit, Catholic University of Health and Allied Sciences, P.O. Box 1464, Bugando, Mwanza, Tanzania; ^2^Tropenmedizin, Missionsärztliche Klinik, Salvatorstr. 7, 97074 Würzburg, Germany; ^3^Medical Microbiology, Goettingen, Germany; ^4^Medical Mission Institute, Hermann Schell Str. 7, 97074 Würzburg, Germany; ^5^Department of Microbiology and Immunology, Catholic University of Health and Allied Sciences, P.O. Box 1464, Bugando, Mwanza, Tanzania

## Abstract

*Objectives. Helicobacter pylori* (*H. pylori*) isolates resistant to clarithromycin and quinolones are increasing worldwide. Data regarding the magnitude of* H. pylori* resistance are limited in developing countries. Here, we report the prevalence of mutations conferring resistance to clarithromycin and fluoroquinolones among dyspeptic patients attending a tertiary hospital, Tanzania.* Methods.* Between August 2014 and August 2016, patients undergoing upper gastrointestinal endoscopy at the Bugando Medical Centre were enrolled. Biopsies were taken for polymerase chain reaction (PCR) and sequencing to detect mutations conferring resistance to clarithromycin and fluoroquinolones.* Results.* A total of 208 nonrepetitive biopsies were examined of which 188 (90.4%) tested positive for* H. pylori* specific 23S rRNA PCR. Clarithromycin resistance mutations were detected in 54/188 (28.7%) of patients tested. The most frequently detected mutation was A2143G (30) followed by A2142G (20). Out of 131 nonrepetitive biopsies tested for fluoroquinolones resistance mutations, 77/131 (58.8%) were positive, with N87I (20) mutation being the most frequently detected mutation followed by A92T mutation which was detected in 16 samples.* Conclusion.* A significant proportion of dyspeptic patients attending tertiary hospital in Tanzania are infected with* H. pylori* strains harbouring clarithromycin or fluoroquinolones resistance mutations. Detection of more than 50% of strains with fluoroquinolones resistance mutations makes the* H. pylori* second line treatment questionable in our setting. There is a need of surveillance of* H. pylori* resistance patterns in Tanzania to provide data that can guide empirical treatment to reduce associated morbidity of* H. pylori* infections. The correlation between A92T fluoroquinolone mutation and phenotypic resistance requires further investigations.

## 1. Introduction

*H. pylori *is a gram negative bacterium, spiral shaped, microaerophilic, and motile with polar flagella, belonging to the genus* Helicobacter*.* H. pylori* can lead to gastritis, peptic ulcer diseases, and gastric cancers [1]. Invasive and noninvasive tests can be used to diagnose* H. pylori* infection; however, culture and molecular tests are methods which can detect the presence of organism as well as the resistance patterns of the* H. pylori* strains [2]. Polymerase chain reaction (PCR), which selectively amplifies the target gene, is a quick,, highly sensitive, and specific test to diagnose* H. pylori *infections [3]. In developing countries, culture and molecular methods are not routinely done. Molecular method is among the methods used for the detection of* H. pylori* and determination of mutation which confers antimicrobial resistance even at a concentration so low that could not be detected by the culture [4].

*H. pylori* infection can complicate the chronic atrophic gastritis which is the precancerous stage to adenocarcinoma [5, 6]. In the recent published global cancer statistics, gastric cancer was ranked third for cancer-related mortality worldwide and fifth for incidence [7].* H. pylori* eradication prevents and slows down the progression of nonatrophic chronic gastritis to atrophic gastritis, hence reducing gastric cancer risk [8]. Therefore, early treatment of patients with* H. pylori* may decrease gastric cancer incidence and its associated mortality [8].

The treatment of* H. pylori* consists of triple therapy (PPI + clarithromycin + either amoxicillin or metronidazole) which can be used in areas with known low clarithromycin resistance, while nonbismuth quadruple concomitant regimen (a proton pump inhibitor, amoxicillin, metronidazole, and clarithromycin) is recommended as first line together with bismuth quadruple therapy (PPI + bismuth + metronidazole + tetracycline) in areas of high clarithromycin resistance [9–11]. Efficacy of these regimens is compromised by drug resistance which is increasing in Africa [12].

Clarithromycin resistance in the first line triple therapy regimens is the main cause of* H. pylori* eradication failure [13, 14]. In the* H. pylori* treatment, clarithromycin is one of the important drugs in the standard therapy of* H. pylori*, while the quinolone is the key drug in the second line therapy [15]. Worldwide, the prevalence of clarithromycin and fluoroquinolone resistance is 19.74% and 18.94%, respectively while in Africa the prevalence of clarithromycin has been found to range from 0% to 100% and that of fluoroquinolones from 0% to 32% [12, 16].

Several mutations have been detected at peptidyl transferase-encoding region in V domain of the* H. pylori* 23S rRNA, which is a component of the large subunit (50S) of the bacterial ribosome. This domain is the most common binding site for antibiotics that inhibit translation like clarithromycin. Therefore, 23SrRNA is used to diagnose* H. pylori* and at the same time detect antibiotic resistance (mutations associated with antibiotic resistance). Most of the known point mutations are A to G transition mutations [17, 18] but three point mutations, namely, A2142G, A2143G, and A2142C, are responsible for 90% of primary clarithromycin resistance in* H. pylori* [19]. In the presence of clarithromycin resistance, eradication failure occurs in about 44.5% to 82% of cases [19]. Second line regimen includes quinolone based therapy which is alternative after failure of first line regimen. Quinolone resistance to* H. pylori* has been associated with second line treatment failure in 27% of patients [20]. A mutation in the quinolone-resistance-determining region (QRDR) is responsible for the resistance to quinolones. Mutations which have been found to cause quinolones resistance include N87H, N87I, N87K, N87Y, D91A, D91G, D91N, and D91Y [12, 21, 22]. The N87 mutations are the key determinants in the failure of quinolones-containing regimen [23].

In Tanzania, most of the first line regimens include clarithromycin while second line regimens are based on quinolones. Despite the high prevalence of* H. pylori* gastroduodenal diseases in Tanzania and the observed high rate of treatment failure, the* H. pylori *mutations conferring resistance to antibiotics have never been studied. Therefore, for the first time in Tanzania, this study has documented the magnitude of* H. pylori* mutations conferring resistance to clarithromycin and fluoroquinolones among dyspeptic patients attending tertiary hospital. These data are highly needed in order to review empirical treatment of* H. pylori* in our setting.

## 2. Materials and Methods

### 2.1. Study Design and Study Population

This was a cross-section study among dyspeptic patients undergoing upper gastrointestinal (GI) endoscopy at the endoscopy unit of the Bugando Medical Centre, from August 2014 to August 2016. All adult dyspeptic patients referred for upper GI endoscopy as part of their workup for their dyspeptic symptoms with no history of antibiotic treatment for* H. pylori* within the past 30 days were included in the study. Dyspepsia was defined according to the ROME criteria [24]. During the upper GI endoscope procedure from one patient, two biopsies were taken from both antrum and fundus. Biopsies for every patient were stored in a single container with 70% ethanol. A total of 208 tissue samples were obtained from 208 patients.

### 2.2. DNA Extraction, Amplification, and Sequencing of the Clarithromycin and Quinolone Resistance-Determining Regions

*DNA Purification from Tissues*. Two biopsies (antrum and fundus) were ground using a tissue homogenizer (UltraTurax; Labo-Moderne, Paris, France). The genomic DNA was extracted using a QIAamp DNA minitissue extraction (Qiagen SA, Courtaboeuf, France) according to the manufacturer's instructions [25].

*H. pylori *detection and clarithromycin mutations: amplification of a 267 bp fragment of the* H. pylori* 23S rRNA was performed by Real-Time PCR (Light Cycler- Roche) using oligonucleotides HPY-S: 5-AGGTTAAGAGGATGCGTCAGTC and HPY-A: 5-CGCATGATATTCCCATTAGCAGT (GenBank accession no. U27270) as previously described [26]. The PCR was carried out in 15*μ*l volume containing Ampli Taq DNA polymerase 1U, PCR buffer 1X, deoxynucleoside triphosphate (dNTP) 200*μ*M, PCR water 7*μ*l, and 0.2*μ*M of each of the primers [26]. After an initial denaturation step at 95°C for 10 minutes, 40 PCR cycles were performed with 95°C for 10 seconds (denaturation), 60°C for 10 seconds (annealing), and 72°C for 20 seconds (extension). Melting curve analysis was performed for each sample. PCR products were purified using the Qiagen PCR-purification kit [25] and sequenced (Seqlab, Göttingen). Mutations within the 23S gene were detected by DNA sequence alignment with the wild type allele using the Geneious software package [version 8.0.4 available from www.geneious.com (Biomatters, Ltd.)].

*GyrA Genes Amplification for Fluoroquinolones Mutations.* Using oligonucleotides GyrA-1 (TTAGCTTATTCAATGAGCGT) and GyrA-2 (GCAGACGGCTTGGTAGAATA), a 428 bp* Gyr*A fragment was amplified from genomic DNA by Real-Time PCR (Light Cycler, Roche) as previously described [27]. The PCR was carried out in 20*μ*l volume containing 4 *μ* (Ampli Taq DNA polymerase 1U, PCR buffer 1X, deoxynucleoside triphosphate (dNTP) 200*μ*M), 5 *μ*l DNA, 7 *μ*l H_2_O, and 2 *μ*l of each primer (5 *μ*M). After an initial denaturation step at 95°C for 10 minutes, 40 PCR cycles were performed with 95°C for 10 seconds (denaturation), 55°C for 10 seconds (annealing), and 72°C for 20 seconds (extension). PCR products were purified using the Qiagen PCR-purification kit [25] and subjected to DNA sequencing (Seqlab, Göttingen). Mutations within the quinolone resistance-determining region (QRDR) of the* H. pylori* GyrA gene (GenBank accession no. AE000583) [28] were detected by DNA sequence alignment with the wild type allele using the Geneious software package [version 8.0.4 available from www.geneious.com (Biomatters, Ltd.)].

This molecular work was done in the Department of Medical Microbiology, University of Gottingen, Germany. Data were entered in Excel sheet and summarized using percentages. The total numbers of samples tested for clarithromycin and fluoroquinolones mutations were used as denominators.

## 3. Results

**Figure 1:**
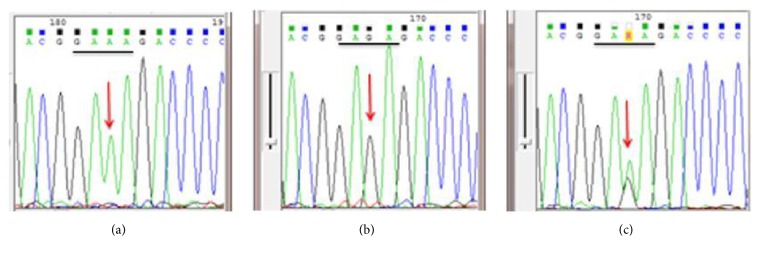


Out of 208 biopsies from nonrepetitive patients examined for* H. pylori* by PCR method, 188/208 (92.2%) were PCR positive. Mutations conferring resistance to clarithromycin were detected in 54/188 (28.7%) of patients. The mutations detected were A2143G (30) {Figure 1}, A2142G (20), A2142C (1), and A2143C (1). Two samples had double mutations A2142G +A2143G and 22/188 (11.7%) samples had both wild type and mutants (Table 1).

**Figure 2:**
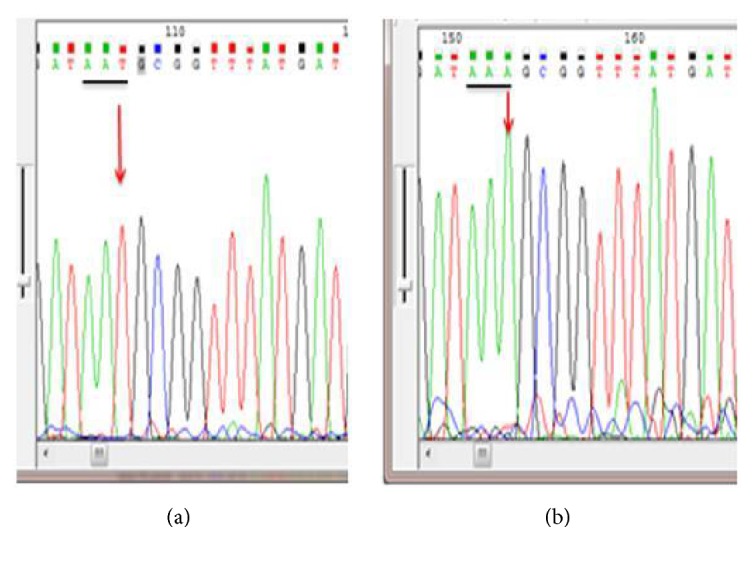


On the other hand, out of 188 patients with positive* H. pylori* PCR, 131 (69.7%) were analyzed for* gyr*A mutations that are known to confer fluoroquinolones resistance. Fluoroquinolone resistance mutations were detected in 77/131 (58.8%) samples; these included N87I (20) {Figure 2}, N87K (7), D91G (8), D91N (15), D91Y (11), and A92T (20.8%) Table 2. Nine (11.7%) samples out of 77 had both wild type and mutants or had heterozygote* H. pylori* strains (Table 2).

A total of 20/77 (25.9%) who had mutation in gyrA gene had also point mutation in* H. pylori* 23S rRNA gene implying that 20/54(37%) of samples with clarithromycin mutations had also quinolones mutations.

## 4. Discussion

Worldwide, the prevalence of primary* H. pylori* resistant to clarithromycin is 19.4% [16]. In European countries, a high prevalence has been reported, ranging from 12.5% to 23.5% [19], while in Africa the overall clarithromycin resistance was 29.2% [12]. In our study, we have observed the presence of clarithromycin mutations that predict drug resistance in 28.7% of patients who were not on eradication therapy. These findings are in line with other studies [12, 29, 30]. However, the observed prevalence of clarithromycin mutations is low compared to the prevalence found in certain parts of Asia (Ina and Vietnam), whereby the prevalence of 43% and that of 85.5%, respectively, were observed [31, 32], and it is higher compared to study done in Congo Brazzaville [33]. The high prevalence of clarithromycin mutations in developing countries could be linked to the overuse of macrolides for treatment of diarrheal diseases in developing countries [34].

In 23S rRNA gene, most of the known point mutations conferring resistance to clarithromycin are A to G transition mutations [18]. Of these, three point mutations, namely, A2142G, A2143G, and A2142C, are responsible for 90% of primary clarithromycin resistance in* H. pylori* [19]. This was confirmed in the present study, whereby A2143G, A2142G, and A2142C formed the majority of clarithromycin mutations detected [17, 19, 35, 36]. It should be noted that the observed mutations have different therapeutic outcome; the presence of A2143G significantly reduced the eradication rate of the* H. pylori* compared to other mutations [36, 37]. Of note, clarithromycin resistance mutations detected in this cohort significantly predicted treatment failure as documented in our previous publication [38]. In areas with high prevalence of clarithromycin resistance of >15%, the recommended regimens are the Bismuth quadruple therapy and concomitant therapy for 14 days according to the Canadian guidelines and 10 days according to the North American and European guidelines [9, 11, 15]. Other mutations which can be found in other parts of the world which have been found to confer clarithromycin resistance in* H pylori* strains are the T2289C, T2190C, T2182C, A2223G, C2195T, C2245T, C2694A, G2141A and G2224A, A2146C, A2146G, and A2147G [39–42].

Regarding fluoroquinolones resistance rates, 3.9% prevalence has been reported in Europe and 17.4% in Africa [43]. The highest rate in Africa has been reported in Congo Brazzaville [12, 33, 44]; however in the current study, about 59% of patients studied carried known fluoroquinolones resistance mutations. Quinolone resistance to* H. pylori* has been associated with second line treatment failure [20]; therefore these findings are alarming because a significant proportion of patients on the second line regimen might have treatment failure. This could be explained by the fact that in Tanzania quinolones are commonly used in the treatment of urinary tract infections, typhoid fever, infectious diarrhea, and genital discharge syndrome, hence selecting for* H. pylori *resistant strains. The alternative for treatment of* H. pylori* for the patients who fail second line is bismuth quadruple therapy [45] which is not commonly available in lower health facilities.

The main fluoroquinolones resistance mutations have been detected in* gyr*A gene at the codon positions 87, 88, 91, and 97 [46, 47]. As in other studies, in the current study the commonest mutations were in gyr87 [12]. In our study, the mutation A92T in the* gyr*A gene was detected for the first time in* H. pylori*. This mutation has been reported in* Neisseria gonorrhea *[48]. In that study, the resistant isolates to gepotidacin (topoisomerase type II inhibitor) were found to have an additional A92T mutation. In our study, other mutations which have been identified in other parts of the world were not detected; these mutations include N87H, N87Y, and D91A. This could be explained by the fact that the distribution of mutations depends on the phylogeographic tree differences of* H. pylori* due to gene content diversity which can be due to either gene loss or gene recombination in multiple strains [49].

## 5. Conclusion

A significant proportion of dyspeptic patients attending tertiary hospital in Tanzania are infected with* H. pylori* strains harboring clarithromycin or fluoroquinolones resistance mutations. Detection of more than 50% of strains with fluoroquinolones resistance mutations makes the* H. pylori* second line treatment questionable in our setting. There is a need of surveillance of* H. pylori* resistance patterns in Tanzania to provide data that can guide empirical treatment to reduce associated morbidity and mortality of* H. pylori* infections. The correlation between A92T fluoroquinolone mutation and phenotypic resistance in* H. pylori* requires further investigations

## Figures and Tables

**Table 1:** Patterns of mutations among 54 patients/samples with point mutations.

Antibiotic tested	Tested samples	Mutation type	All point mutations identified	Heterozygote *H. pylori* strains	Homozygote *H. pylori* *strains*
Clarithromycin	188	A2143G	30/54	13/30	17/30
		A2142G	20/54	14/20	6/20
		A2142C	1/54	0	1
		A2143C	1/54	0	1
		A2143G + A2142G	2/54	1/2	1/2

**Table 2:** Patterns of mutation among 77 patients/samples with point mutations.

Antibiotic tested	Tested samples	Mutation type	All point mutations identified	Heterozygote *H. pylori* strains	Homozygote *H. pylori* *strains*
Fluoroquinolones	131	N87I	20	5/20	15/20
		N87K	7	3/7	4/7
		D91G	8	2/8	6/8
		D91N	15	1/15	14/15
		D91Y	11	1/11	10/11
		A92T*∗*	16	2/16	14/16

*∗*Unknown mutation

## Data Availability

The data belongs to the CUHAS University and Bugando Hospital; a permission is required to make them freely available.

## Additional Points

*Highlights.* (i) 90% PCR positive for* H. pylori.* (ii) High prevalence of* gyr*A mutations. (iii) High frequency of A2142G and N87I. (iv) A92T unknown mutation in* gyr*A gene.
